# Supplementary material for: Clinical Relevance of VEGFA (rs3025039) +936 C>T Polymorphism in Primary Myelofibrosis: Susceptibility, Clinical Co-Variates, and Outcomes
Source: Genes (Basel). 2021 Aug 20;12(8):1271. doi: 10.3390/genes12081271 (PMC8393853; doi:10.3390/genes12081271)
Supplement: Supplementary file 1 [file genes-12-01271-s001.zip › genes-1293972-supplementary.pdf]

Table **S1.** Frequency of the genotypes and of the T-allele of the *VEGFA* rs3025039 polymorphism in subjects with primary myelofibrosis (PMF) stratified according to the somatic driver mutations

	Numbers	***VEGFA rs3025039* genotype, N (% of PMF subjects)**					**Genotype frequencies**		**T-allele frequency**		
		CC	CT	TT	CC /CT	CT/TT	CT/TT vs. CC OR (95% CI)	TT vs. CC/CT OR (95% CI)	T-allele frequency (%)	vs. total PMF population	vs healthy population
PMF, all subjects	827	584	219	24	803	243			267/1654 (16.1)		
JAK2^{V617F}-positive, N (%)	544 (65.8)	367 (62.8)	161 (73.5)	16 (66.6)	528 (65.7)	177 (72.8)	**OR = 1.58 (1.41-2.20) *P* = 0.006**	OR = 1.04 (0.44-2.46) *P* = 0.92	193/1088 (17.7)	OR = 1.12 (0.91, 1.37) *P* = 0.27	OR = 0.97 (0.73, 1.28) *P* = 0.82
JAK2^{V617F}-negative, N (%)	283 (34.2)	217 (36.2)	58 (26.5)	8 (33.3)	275 (34.2)	66 (27.2)	**OR = 0.63 (0.45-0.87) *P* = 0.006**	OR = 0.96 (0.40-2.27) P=0.92	74/566 (13.1)	OR = 0.78 (0.59, 1.03) *P* = 0.08	**OR = 0.68 (0.48, 0.94) *P* = 0.021**
CALR mutation, N (%)	171 (20.7)	130 (22.2)	36 (16.4)	5 (20.8)	166 (20.7)	41 (16.9)	OR =0.70 (0.48-1.04) P = 0.08	OR =1.00 (0.37-2.74) *P* = 0.98	46/342 (13.4)	OR = 0.80 (0.58, 1.13) *P* = 0.21	OR = 0.70 (0.47, 1.03) *P* = 0.07
MPL mutation, N (%)	44 (5.3)	35 (6.4)	9 (4.1)	0 (0)	44 (5.5)	9 (3.7)	OR = 0.56 (0.26-1.19) *P* = 0.13	OR = 0.35 (0.02-5.82) *P* = 0.43	9/88 (10.2)	OR = 0.59 (0.29, 1.19) *P* = 0.14	OR = 0.51 (0.24, 1.06) *P* = 0.07
Triple negative, N (%)	68 (8.2)	52 (9.5)	13 (5.9)	3 (12.5)	65 (8.1)	16 (6.6)	OR = 0.67 (0.35, 1.20) *P* = 0.18	OR = 1.62 (0.47, 5.58) *P* = 0.44	19/136 (14)	OR = 0.84 (0.51, 1.39) *P* = 0.50	OR = 0.73 (0.42, 1.24) *P* = 0.25

Table S2. Clinical and laboratory co-variates by *VEGFA* rs3025039 genotype in subjects with primary myelofibrosis (PMF).

	**N**	**Genotype**					**Comparison of genotype frequencies**	
		CC	CT	TT	CC /CT	CT/TT	CT/TT vs. CC.	TT vs. CC/CT
	849	601	224	24	825	248		
**Demographic co-variates**								
Age at diagnosis, (yrs, median (IQR)	52 (40-61)	52 (39-61)	52 (41-61)	51 (39-62)	52 (40-61)	52 (41-62)	P = 0.45	P = 0.92
Sex, males, N (% of the total PMF population)	503 (59.2)	350 (58.2)	139 (62.0)	14 (58.3)	489 (97.2)	153 (61.7)	OR =1.15 (0.85, 1.56) P = 0.35	OR =0.96 (0.42, 2.19) P = 0.92
**Laboratory co-variates**								
Hemoglobin, g/L; median (IQR)	131 (109-148)	131 (110-147)	130 (108-150)	119 (90.5-139)	131 (110-148)	130 (106-149)	P = 0.73	P = 0.06
WBC x10E+9/L; median (IQR)*	8.5 (6.4-11.5)	8.4 (6.6-11)	8.7 (6.4-12)	8.5 (5-10)	8.5 (6.4-12)	8.7 (6.3-12)	P = 0.96	P = 0.46
Platelets x 10E+9/L; median (IQR)	467 (246-711)	479 (251-718)	459 (239-406)	281 (116-587)	474 (249-714)	450 (215-679)	P = 0.26	**P = 0.023**
Platelets <150 x 10E+9/L (%)	121/839 (14.4)	78/594 (13.1)	35/221 (15.8)	8/24 (33.3)	113/815 (13.8)	43/245 (17.5)	OR = 1.40 (0.94, 2.11) P = 0.09	**OR = 3.10 (1.29, 7.42) P = 0.011**
Spleen index, cm x E+2; median (IQR)	120 (90-160)	120 (90-150)	120 (90-180)	125 (90-169)	120 (90-160)	120 (90-180)	P = 0.12	P = 0.53
Monocytes x 10E+9/L, N, median (IQR)	456 496 (334-688)	335 499 (337-686)	106 486 (319-713)	15 483 (240-784)	441 498 (336-688)	121 483 (297-646)	P = 0.91	P = 0.84
Serum lactate	469	330	127	12	457	139	P = 0.07	**P = 0.044**

dehydrogenase (LDH) level x ULN, N, median (IQR)	1.28 (0.92-1.94)	1.20 (0.87-1.94)	1.35 (1-1.07)	1.58 (1.35-2.51)	1.26 (0.91-1.94)	1.36 (1-1.97)		
LDH (ULN>1), N (%)	316 (67.4)	213 (64.5)	91 (71.6)	12 (100)	304 (66.5)	103 (74.1)	OR = 1.57 (1.10, 2.44) *P* = 0.044	OR = 12.6 (0.74, 2.14) *P* = 0.076
**Biological co-variates**								
CD34 positive cells in PB, x10E+6/L; N, median (IQR)	401 10 (4-43)	285 8 (4-34)	100 11 (5-72)	16 18 (3-53)	385 9 (4-39)	119 12 (5-68)	*P* = 0.12	*P* = 0.82
Serum cholesterol, mg/dL; N, median (IQR)	420 159 (129-183)	298 162 (133-185)	110 150 (127-180)	12 140 (118-166)	408 159 (130-184)	122 147 (124-180)	*P* = 0.06	*P* = 0.14
NGS detected mutations N. (%)	465/242 (19)	30/172 (17)	16/65 (25)	0/5 (0)	46/237 (19)	16/70 (23)	OR = 1.44 (0.72, 2.86) *P* = 0.29	OR = 2.67 (0.14, 48.4) *P* = 0.51
Cytogenetic abnormalities N (%)	86/290 (29.6)	56/203 (27.6)	25/77 (32.5)	5/10 (50)	81/280 (28.9)	30/87 (34.5)	OR = 1.38 (0.80, 2.36) *P* = 0.24	OR = 2.45 (0.69, 8.7) *P* = 0.16
hs-CRP, ng/ml; N, median (IQR)	237 0.15 (0.05-0.63)	169 0.14 (0.05-0.57)	59 0.19 (0.10-0.92)	9 0.15 (0.06-0.55)	228 0.15 (0.05-0.64)	68 0.18 (0.10-0.80)	*P* = 0.07	*P* = 0.99
CD34/CXCR4 %, N, median (IQR)	297 41 (21-63)	205 41 (21-64)	80 40 (20-53)	12 46 (25-64)	285 41 (21-63)	92 41 (20-54)	*P* = 0.29	*P* = 0.82
BM fibrosis grade 0-1 N (%)	484/846 (57.2)	355/598 (59.4)	120/224 (53.6)	9/24 (37.5)	475/822 (57.8)	129/248 (52)	**OR = 0.74 (0.55, 0.99) *P* = 0.05**	**OR =0.44 (0.18, 1.01) *P* = 0.05**
BM fibrosis grade 2-3 N (%)	362/846 (42.8)	243/598 (40.6)	104/224 (46.4)	15/24 (62.5)	347/822 (42.2)	119/248 (48)	**OR = 1.34 (1.00, 1.81) *P* = 0.05**	**OR = 2.28 (0.98, 5.27) *P* = 0.05**

Table S3. Clinical and laboratory co-variates of PMF subjects with *JAK2*^{V617F} and *VEGFA* rs3025039 genotype

	N	Genotype					Comparison between genotypes	
		CC	CT	TT	CC /CT	CT/TT	CT/TT vs.CC	TT vs. CC/CT
Number	544	367	161	16	528	177		
**Clinical and hematologic characteristics**								
Hemoglobin, g/L; median (IQR)	13.7 (11.6-15.5)	13.8 (11.8-15.5)	13.6 (11.5-15.5)	13.4 (10.1-14.4)	13.7 (11.6-15.5)	13.5 (11.4-15.5)	*P* = 0.36	*P* = 0.10
WBC, x 10 E+9/L; median (IQR)	9.1 (6.7-12.2)	9.1 (6.8-12.4)	9.0 (6.7-12)	9.1 (5.4-11.6)	9.1 (6.8-12.2)	9.0 (6.7-12)	*P* = 0.68	*P* = 0.74
Platelets x 10 E+9/L; median (IQR)	450 (248-655)	473 (264-660)	416 (215-646)	281 (139-442)	455 (251-655)	394 (205-638)	*P* = 0.09	***P* = 0.033**
Platelets < 150 x10E+9/L, N, (%)	79/536 (14.7)	46/362 (12.7)	29/158 (18.3)	4/16 (25)	75/520 (14.4)	33/174 (18.9)	OR =1.60 (0.98, 2.62) *P* = 0.06	OR =1.97 (0.62, 6,29) *P* = 0.25
Spleen index, cm x E+2; median (IQR)	120 (90-176)	120 (90-170)	120 (90-180)	125 (115-169)	120 (90-176)	120 (90-180)	*P* = 0.36	*P* = 0.64
Monocytes x10E+9/L, N, median (IQR)	286 488 (328-688)	199 490 (337-688)	76 477 (297-679)	11 340 (240-822)	275 490 (335-688)	87 473 (279-688)	*P* = 0.68	*P* = 0.45
Serum lactate dehydrogenase level x ULN, N, median (IQR)	308 1.15 (0.88-1.75)	207 1.14 (0.84-1.69)	88 1.19 (0.95-1.76)	13 1.79 (1.40-3.20)	295 1.14 (0.87-1.73)	101 1.29 (0.98-1.81)	*P* = 0.08	***P* = 0.005**
**Biological characteristics**								
Blood CD34-positive cells, x10E+6/L; N, median (IQR)	259 7.5 (3.7-160)	180 6.3 (3.6-175)	69 9.4 (3.9-33)	10 28.5 (18-47)	249 7.1 (3.7-26.5)	79 11.1 (4.4-45)	*P* = 0.07	***P* = 0.027**
Serum cholesterol,	267	182	77	8	259	85	***P* = 0.046**	P = 0.24

mg/dL; N, median (IQR)	156 (129-179)	160 (133-180)	146 (124-178)	147 (118-151)	157 (129-180)	146 (122-176)		
NGS mutations N. (%)	20/148 (13.5)	12/101 (11.9)	8/45 (17.8)	0/2 (0)	20/146 (13.7)	8/47 17.0	OR = 1.52 0.57, 4.01 *P* = 0.39	1.23 (0.58, 26.41) *P* = 0.89
Cytogenetic abnormalities N (%)	61/190 (32.1)	36/126 (28.6)	20/56 (37.5)	5/8 (62.5)	56/182 (30.8)	25/64 (39)	OR =1.60 (0.85, 3.02 *P* = 0.14	OR = 3.75 (0.86, 16.23 *P* = 0.08
hs-CRP, ng/ml; N, median (IQR)	150 0.14 (0.06-0.57)	106 0.12 (0.05-0.56)	39 0.34 (0.11-0.92)	5 0.15 (0.10-0.31)	145 0.14 (0.06-0.57)	44 0.31 (0.10-0.87)	***P* = 0.018**	*P* = 0.83
CD34/CXCR4 %, N, median (IQR)	188 43 (23-63)	125 42 (24-65)	55 41 (20-54)	8 45 (16-57)	180 42 (23-63)	63 43 (19-54)	*P* = 0.44	*P* = 0.63
BM fibrosis grade, 0-1; N (%)	335/542 (61.8)	234/365 (64.1)	94/161 (58.4)	7/16 (43.7)	328/526 (62.3)	101/177 (57.1)	OR = 0.74 (0.51, 1.07) *P* = 0.11	OR = 0.47 (0.17, 1.28) *P* = 0.14

IQR = interquartile range; PMF = primary myelofibrosis; NGS = next generation sequencing; CRP = C-reactive protein

Table S4. Clinical and laboratory co-variates of PMF subjects without *JAK2*^{V617F} and *VEGFA* rs3025039 genotype.

	Number	Genotype					Comparison between genotypes	
		CC	CT	TT	CC /CT	CT/TT	CT/TT vs. CC	TT vs. CC/CT
	283	217	58	8	275	66		
**Clinical and hematologic characteristics**								
Hemoglobin, g/L; median (IQR)	121 (102-136)	123 (104-136)	120 (99-137)	100 (89-126)	122 (104-136)	118 (98-135)	*P* = 0.42	*P* = 0.18
White-blood cell count, x 10E+9/L; median (IQR)	7.7 (6.0-9.9)	7.7 (6-9.9)	7.3 (6.0-10.4)	6.5 (4.2-9.0)	7.7 (6.0-9.9)	7.3 (5.7-10.1)	*P* = 0.82	*P* = 0.30
Platelet count, x 10E+9/L; median (IQR)	510 (245-775)	490 (235-775)	597 (320-822)	409 (101-724)	513 (245-776)	588 (291-763)	*P* = 0.42	*P* = 0.31
Spleen index, cm x E+2; median (IQR)	110 (90-140)	100 (90-135)	113 (90-150)	110 (90-170)	110 (90-140)	113 (90-150)	*P* = 0.36	*P* = 0.87
Monocyte count, x10E+9/L, N, median (IQR)	162 507 (336-702)	127 504 (336-679)	29 520 (330-891)	6 595 (395-784)	156 506 (334-693)	35 531 (330-891)	*P* = 0.39	*P* = 0.46
Serum lactate dehydrogenase level x ULN, N, median (IQR)	157 1.58 (1.1-2.3)	117 1.54 (1-2.27)	36 1.78 (1.34-2.36)	4 1.46 (1.31-2.75)	153 1.61 (1.06-2.3)	40 1.68 (1.34-2.36)	*P* = 0.19	*P* = 0.74
**Biological characteristics**								
Blood CD34-positive cells, x 10 E+6/L; median (IQR)	133 19 (6-68)	98 18 (6-60)	28 52 (8-80)	7 3 (2-46)	126 21 (6-72)	35 38 (5-77)	*P* = 0.37	*P* = 0.06
Serum cholesterol, mg/dL; N, median (IQR)	147 163 (131-192)	109 163 (135-193)	33 166 (131-183)	5 135 (123-181)	142 163 (133-192)	38 163 (128-183)	*P* = 0.62	*P* = 0.38

hs-CRP, ng/ml; N, median (IQR)	82<br>0.17<br>(0.04-0.7)	59<br>0.02<br>(0.05-1.79)	19<br>0.18<br>(0.04-0.74)	4<br>0.30<br>(0.04-0.63)	78<br>0.17<br>(0.04-0.70)	23<br>0.18<br>(0.04-0.72)	*P* = 0.79	*P* = 0.92
CD34/CXCR4 %, N, median (IQR)	104<br>40<br>(18-63)	75<br>40<br>(18-66)	24<br>31<br>(19-49)	5<br>48<br>(41-67)	99<br>37<br>(18-63)	29<br>41<br>(21-50)	*P* = 0.70	*P* = 0.29
BM fibrosis grade, 0-1; N (%)	137/282<br>(48.6)	112/216<br>(51.8)	23/58<br>(39.6)	2/8<br>(25)	135/274<br>(49.3)	25/66<br>(37.9)	OR = 0.56<br>(0.32-0.99)<br>*P* = 0.048	OR = 0.34<br>(0.07-1.73)<br>*P* = 0.19

IQR = interquartile range; PMF = primary myelofibrosis; NGS = next generation sequencing; CRP = C-reactive protein

Table S5. Hazard ratio (HR) of the outcomes of PMF subjects with *JAK2*^{V617F} and *VEGFA* rs3025039 genotypes.

	**CT/TT (N=177) *vs*. CC (N=367)**		**TT (N=16) *vs*. CC/CT (N=528)**	
	HR (95% CI)	*P*-value	HR (95% CI)	*P*-value
Hemoglobin less than 100 g/L	1.19 (0.91, 1.59)	0.22	1.49 (0.73, 3.03)	0.27
Spleen > 10 cm from the left costal margin	1.31 (1.01,1.72)	**0.05**	1.01 (0.45, 2.27)	0.98
WBC > 12 x 10E+9/L	1.05 (0.81, 1.37)	0.73	1.04 (0.49, 2.22)	0.90
WBC < 4 x 10E+9/L	1.30 (0.79, 2.13)	0.31	1.83 (0.25, 13.2)	0.54
Platelets < 150 x 10E+9/L	1.25 (0.91, 1.72)	0.17	2.56 (1.35, 5)	**0.004**
Blood CD34-positive cells >100 x 10E+6/L	1.31 (0.93, 1.85)	0.12	1.72 (0.69, 4.16)	0.24
Transplant	1.78 (0.98, 3.22)	0.06	1.75 (0.42, 7.14)	0.44
Blast transformation	1.09 (0.68, 1.75)	0.72	2 (0.72, 5.55)	0.18
Death	0.30 (0.92, 1.82)	0.14	2.13 (1.03, 4.35)	**0.04**
